# 

**Published:** 1890-10-18

**Authors:** 


PRICE ONE PENNY.

No. 212, Vol. IX.—October 18th, 1890.

[Registered at the General Post Office as a Newspaper for transmission in the United Kingdom and Abroad.]

WITH EXTRA NURSING SUPPLEMENT.

Per Annum, 6s. 6d.; post free.

Monthly Parts, 6d.; post free 8d.

Ditto per Annum, 8s., post free.

### THE HOSPITAL

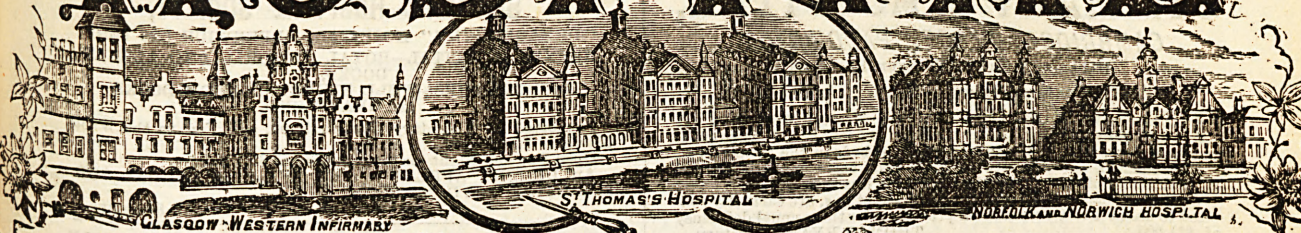

A WEEKLY JOURNAL OF

Science, Medicine, Nursing and Philanthropy.

SATURDAY, OCTOBER 18th, 1890.

**Low Birthrates and Population** . . . . . . 33
**Passing Topics.**—Male Nurses.—I. . . . . . . 34
**The Doctor's Pulpit.**—How to Keep Well . . . . . 35
**Working Women in Large Towns.**—XIII. Some Sources of Distress . . . . . . . . . . 36
**The History and Capabilities of Herbal Simples.**—By W. F. Fernie, M.D.—XXI. Clover—Comfrey . . . . 37
**Turning Over New Leaves.**—The Drink Question . . 38
**The Medical Education of Women** . . . . . 39
**Notes and News** . . . . . . . . . 40
**The Story of the Insane from Year to Year** . . . 41
**Editor's Letter Box.**—"No Gentleman Keeps a Bulldog." . 46
**Annotations.**—Poor Men's Houses—The Hospitals and the Pension Fund—Transplanting a Hospital . . . . 42
**The Practitioner's Mirror and Retrospect:**—
The London Post-Graduate Course at the Paddington Infirmary . . . . . . . . . . 43
Introductory Lecture—What? When? and How? By Jonathan Hutchinson, LL.D., F.R.S. . . . . . 44
**Scraps and Gleanings** . . . . . . . . 46
**The Medical Students' Supplement.**—In the Cause of Medical Students—Promising Students—Students' Medical Societies, 47; Appointments — Scholarships and Prizes—Athletics—Events for the Month of October . . . . 48

### EXTRA SUPPLEMENT—THE NURSING MIRROR.

**En Passant.**—Edinburgh Royal Infirmary—Short Items—Lectures—Cottage Nurses—Miss Kate Marsden—Hope Hospital . . . . . . . . . . . . ix
**Sketches of Insanity.** By F. H. Walmsley, M.D. (conclusion).—XIV. Management of the Insane . . . . . x
**Six Months in a Hospital.**—By a "Special Pro." . . . x
**Southall's Patents—Death in Our Ranks** . . . . xi
**The Nurse's Bookshelf.**—Advice to Mothers—Nursery Health Tracts . . . . . . . . . . . xi
**Examination Questions** . . . . . . . . . xi
**Everybody's Opinion.**—The Nurses of the London Hospital . xii
**Lectures and Amusements—Notes and Queries** . . xii
**Appointments** . . . . . . . . . . xii
**Vacancies—Amusements and Relaxation** . . . . xii

